# Graphing and reporting heterogeneous treatment effects through reference classes

**DOI:** 10.1186/s13063-020-04306-1

**Published:** 2020-05-07

**Authors:** James A. Watson, Chris C. Holmes

**Affiliations:** 1 Mahidol-Oxford Tropical Medicine Research Unit, Faculty of Tropical Medicine, Mahidol University, Bangkok, 10400 Thailand; 2 Centre for Tropical Medicine and Global Health, Nuffield Department of Medicine, University of Oxford, Oxford, UK; 3 Department of Statistics, University of Oxford, Oxford, UK

**Keywords:** Heterogeneous treatment effects, Reference class forecasting, Randomised controlled trials, Prognostic risk score

## Abstract

**Background:**

Exploration and modelling of heterogeneous treatment effects as a function of baseline covariates is an important aspect of precision medicine in randomised controlled trials (RCTs). Randomisation generally guarantees the internal validity of an RCT, but heterogeneity in treatment effect can reduce external validity. Estimation of heterogeneous treatment effects is usually done via a predictive model for individual outcomes, where one searches for interactions between treatment allocation and important patient baseline covariates. However, such models are prone to overfitting and multiple testing and typically demand a transformation of the outcome measurement, for example, from the absolute risk in the original RCT to log-odds of risk in the predictive model.

**Methods:**

We show how reference classes derived from baseline covariates can be used to explore heterogeneous treatment effects via a two-stage approach. We first estimate a risk score which captures on a single dimension some of the heterogeneity in outcomes of the trial population. Heterogeneity in the treatment effect can then be explored via reweighting schemes along this axis of variation. This two-stage approach bypasses the search for interactions with multiple covariates, thus protecting against multiple testing. It also allows for exploration of heterogeneous treatment effects on the original outcome scale of the RCT. This approach would typically be applied to multivariable models of baseline risk to assess the stability of average treatment effects with respect to the distribution of risk in the population studied.

**Case study:**

We illustrate this approach using the single largest randomised treatment trial in severe falciparum malaria and demonstrate how the estimated treatment effect in terms of absolute mortality risk reduction increases considerably in higher risk strata.

**Conclusions:**

‘Local’ and ‘tilting’ reweighting schemes based on ranking patients by baseline risk can be used as a general approach for exploring, graphing and reporting heterogeneity of treatment effect in RCTs.

**Trial registration:**

ISRCTN clinical trials registry: ISRCTN50258054. Prospectively registered on 22 July 2005.

## Background

Randomised controlled trials (RCTs) provide the best causal evidence base for estimating the population average treatment effect (ATE) for a given intervention. The ATE can be used to support optimal decision making at the population level. At the individual level, however, the ATE is often unrepresentative of the true individual treatment effect (ITE) for a large proportion of patients. This may arise when the absolute treatment effect varies as a function of the individual risk of a negative outcome (e.g. treatment failure, death, severe adverse event, etc.). It has been previously shown that the baseline risk of a negative outcome is often highly skewed in patient populations for many diseases and conditions [1]. Hence the average risk, on which the ATE is estimated, may not be a good summary of the individual risk [2].

Due to the common occurrence of heterogeneity in baseline risk, guidelines for the reporting and assessment of RCT results have recommended the use of multivariable risk prediction tools for patient stratification [3–5]. Baseline predicted risk summarises patient variability into a single dimension over which individuals can be compared and average outcomes assessed. In the case that the risk score summarises all of the information content in the baseline covariates regarding the outcome, such that the risk score is sufficient, i.e. the outcome is conditionally independent of the covariates given the risk score, then the risk score is known as a prognostic score [6]. Ideally the risk score is a prognostic score, but this is not necessary for the risk score to be a useful stratifying variable. Risk scores provide a methodology for risk-based reference class forecasting and a principled way of assessing personalised and heterogeneous treatment effects (HTEs) [7]. Here, reference class forecasting refers to a prediction approach whereby each individual is characterised by a univariate (reference) score. We then predict the outcome for a given individual by averaging over the observed outcomes in a set of ‘similar’ individuals [8]. Similarity is defined by the closeness of their reference scores. In our context, we use the baseline risk to define the reference class.

The assessment of HTEs, and thus the stability of trial results, has classically been done by constructing parametric models targeting the ITE, conditioning on either the baseline risk or key variables predictive of the outcome [9]. However, estimating an ITE brings with it two challenges, one foundational and one practical. The foundational challenge is that the estimand of an ITE is counterfactual, targeting the expected difference between an observed (actual) outcome and an unobserved (potential) outcome that would have occurred had the individual been given an alternative treatment. The practical challenge is that statistical methods targeting ITEs invariably need to transform the outcome measurement to allow for contextual modelling, such as transforming to the log-odds scale under logistic regression, or proportional hazards for time-to-event data. These transformations inevitably make results dependent on parametric model assumptions and link functions concerning the effect of patients’ baseline covariates on their outcomes. Moreover, when testing for evidence of clinically significant variation in the ITE, considerable care must be taken not to overfit to the data, especially when considering a large number of potential predictor variables [10]. Overfitting to data can suggest heterogeneity when none exists or can identify spurious associations between covariates and ITEs. Correction for multiple testing and overfitting invariably reduces the power to detect true HTEs. Changing or transforming the outcome measure in order to accommodate modelling assumptions complicates the communication of results if the original trial reports the treatment effect on the absolute scale yet subsequent ITEs are reported on a transformed measure.

Here we promote a non-parametric (model-free) reference class forecasting approach to the estimation and assessment of HTEs using baseline risk to determine the reference class. In particular we construct the reference class through a sample reweighting scheme, and use this to explore for treatment effect variation in target populations different to the one collected through the RCT. Targeted treatment effects can then be estimated for these new populations. Two conceptually different target populations can be defined. Firstly, there is one which allows for the estimation of a counterfactual ITE. This can be done through ‘local’ reference classes centred around an individual of interest. Secondly, there is one which allows the estimation of the ATE in a ‘tilted’ population with different risk distributions to that of the RCT. We denote these as conditional average treatment effects (CATEs). The latter may be particularly useful if the risk profile of the trial population systematically underestimates or overestimates the profile of risk of the population for which the intervention is aimed. The CATE can then be used to explore stability of the RCT outcome with respect to this variation in population risk. The ITE and the CATE correspond to different sample reweighting schemes. We show how different reweighting schemes correspond to different bias-variance trade-offs for the reference class estimator, and we provide guidelines on graphing of results. It is important to note that estimands from reference classes target the same units of treatment effect as those defined in the original RCT. This allows for direct comparison between estimates. This is not true in general for HTE models, which, as noted above, may have to transform the outcome prior to modelling, for example, transforming an absolute effect to the log-odds scale for a binary response. We note that providing individualised predictions through locally weighted averaging has a rich history in the field of kernel smoothing methods, for example, the Nadaraya-Watson estimator [11–13].

For illustration we consider the single largest trial of life-saving interventions in severe malaria, demonstrating that the superiority of parenteral artesunate over parenteral quinine stems from its very large effect in the most severely ill patients.

## Methods

### Multivariable risk-based ranking of trial individuals

In the following we consider RCT data of the form $\{x_{i},y_{i},t_{i}\}_{i=1}^{N}$, where *x*_*i*_ is a vector of baseline patient covariates for the *i*th patient, and *y*_*i*_ is their observed outcome after receiving a randomised treatment allocation indicated by *t*_*i*_. We are interested in pairwise comparisons between two treatment arms *T*_0_ and *T*_1_.

We assume that it is possible to construct a priori a ‘risk quantile mapping’ *Q*:*X*→[0,1] for the outcome *y*_*i*_. This function *Q* maps each subject in the trial to their corresponding empirical risk quantile, agnostic of treatment. Thus, *Q*(*x*_*i*_)=0 denotes the subject least at risk of the negative outcome, and *Q*(*x*_*j*_)=1 the subject most at risk. In practice this mapping could be derived by estimating a function *f*:*X*→*Y* using data from a different source (this could include observational data, as the risk is agnostic of the treatment received); computing *f*(*x*_*i*_) for each subject; and then mapping *f*(*x*_*i*_) onto the empirical risk distribution for the *N* subjects in the trial. For most conditions, there will exist either an already validated risk score or external data on which to build a risk-based ranking [1]. If this is not the case, it is also possible to build an internal risk model by ‘retrodiction’: fitting the function *f* to the trial data at hand. Simulation studies suggest that these internal models introduce little bias into the procedure [14]. We note that a risk quantile mapping can be based on almost any type of outcome data. For example, a proportional hazards (Cox) model fit to time-to-event data can produce a risk quantile mapping by using the estimated linear combination of predictors.

We can then use the risk quantile mapping to construct a reference class. The risk mapping removes the need for multiple testing of interactions between the randomised treatment and single baseline covariates. The use of risk-based reference classes for exploring HTEs has been advocated previously, but it was limited to quintile or quartile subgroups [3–5]. We now consider a general approach using the risk-ranked individuals to estimate ITEs and CATEs. The approach advocated here relies critically on the quality of the risk mapping: the better the quantile mapping (i.e. the better it is at discriminating between low- and high-risk individuals), the better it will be for visualising HTEs if they are present.

In the following, for simplicity we assume that the subject index *i* has subsequently been sorted according to the risk prediction, with *Q*(*x*_1_)=0 and *Q*(*x*_*N*_)=1. In general, and in the absence of ties, $Q(x_{i}) = \frac {i-1}{N-1}$.

### Local smoothing estimation of a risk-based ITE using reference class forecasting

By ranking trial subjects from those ‘least at risk’ to those ‘greatest at risk’, it is possible to use a sliding window approach to estimate the ITE for each subject. For subject *i*, the set of risk-adjacent subjects determines the reference class used to predict the ITE for subject *i*. This generalises the concept of partitioning subjects into quintile or quartile subgroups [11].

**Fig. 1:**
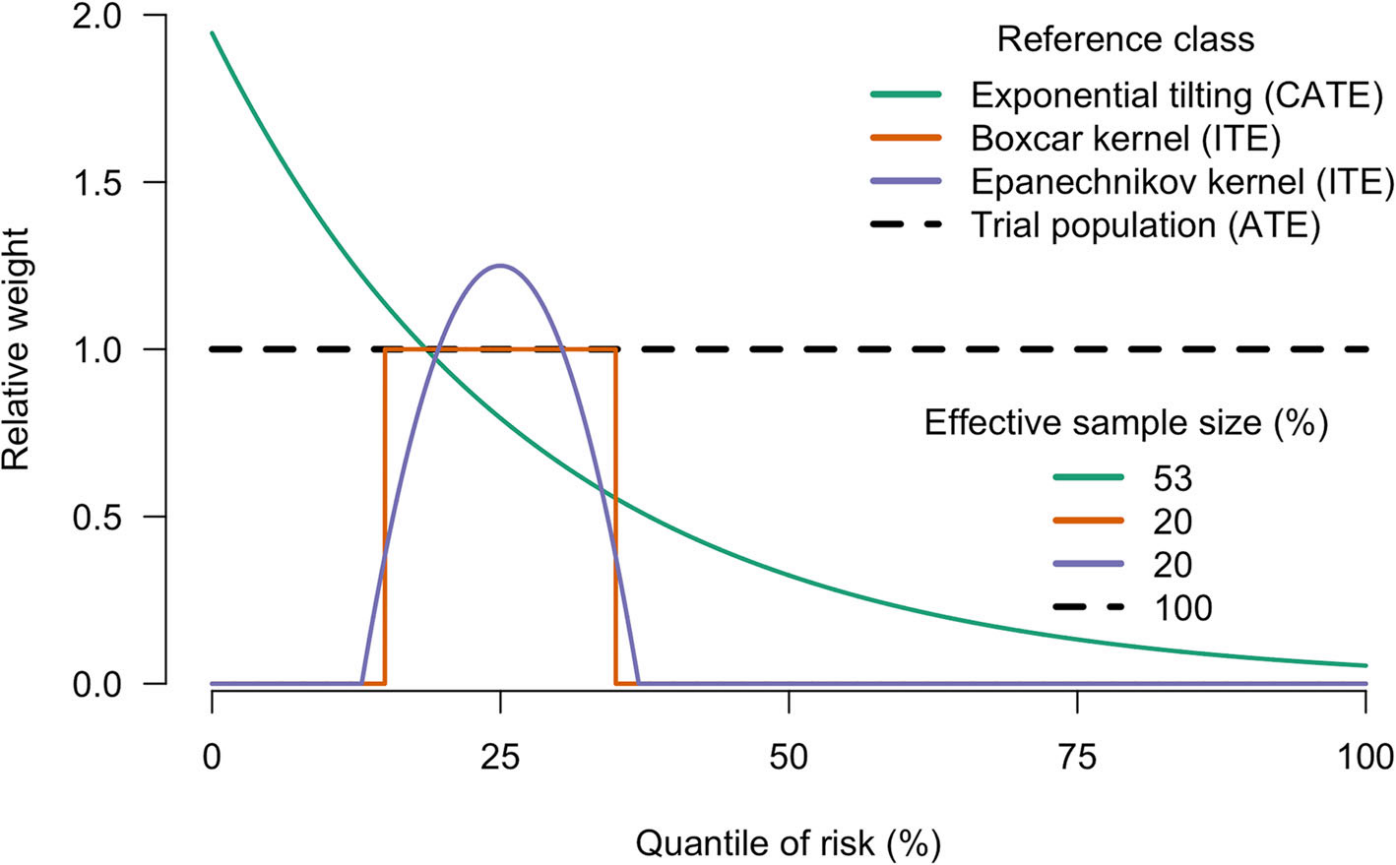
Two local kernel weighting schemes for ITE estimation and one global reweighting scheme for CATE estimation, as compared to ATE estimation. The ITE and CATE reweighting schemes represented all reduce the effective sample size of the original data and target an average risk corresponding to the 25th risk quantile. The scale of the *y*-axis is chosen so that the sum of the weights is equal to the effective sample size, or equivalently that the effective sample size is equal to the area under the curve. The effective sample sizes as a percentage of the original data are shown in the legend

Mathematically, the adjacency can be quantified using localised reweighting kernels. Local kernels target a specific individual focussed at their quantile of risk *q*_*i*_ for the *i*th subject by considering the treatment outcome of other individuals in a local neighbourhood of risk-adjacent individuals, with *q*’s close to *q*_*i*_. These local reference classes are parameterised by their bandwidth (radius) *γ*∈[0,1], which defines the proportion of subjects in the window ‘close’ to subject *i*, which are used to estimate the ITE of the *i*th subject. This in turn characterises the effective sample size of the reference class forecasting method. The simplest local reference class forecasting weighting scheme uses the ‘boxcar’ function that gives equal weight to all subjects in the local window when estimating the ITE (see Fig. 1 for an example). For a window of width *γ*, we can estimate the ITE of treatment *T*_1_ versus *T*_0_ in subject *i* as:

1 $$ {}\text{ITE}(q_{i}) = C \left\{\sum_{k} w_{k} y_{k} \mathbbm{1}(t_{k}=T_{1}) - \sum_{k} w_{k} y_{k} \mathbbm{1}(t_{k}=T_{0}) \right\}  $$

where $C = (\sum _{k} w_{k})^{-1}$ and *w*_*k*_=1 for subjects in the window around subject *i* and *w*_*k*_=0 outside of the window, i.e.:

2 $$ w_{k} = \left\{\begin{array}{ll} 1, & \text{for}\quad i-\lfloor{\gamma/N}\rfloor \leq k \leq i + \lfloor{\gamma/N}\rfloor\\ 0, & \text{otherwise} \end{array}\right.  $$

Equation (1) defines the ITE of subject *i* using outcome data from all subjects whose baseline risk quantile is inside an interval of width 2*γ* centred around the *i*th subject. For the subjects in the lowest and highest risk quantiles, for whom there are not ⌊*γ*/*N*⌋ risk-adjacent subjects on each side, we take the convention to define the ITE as that of the subjects ⌊*γ*/*N*⌋ and *N*−⌊*γ*/*N*⌋, respectively. This convention preserves symmetry at the risk ‘tails’ (highest and lowest risk individuals). We note that *γ* must be large enough such that for each subject *i* there is at least one risk-adjacent subject inside the risk quantile interval of width 2*γ* for both treatment arms (at least one received *T*_0_ and at least one received *T*_1_). Otherwise, the treatment effect is not estimable.

The boxcar kernel is known to be problematic, as it varies in a non-smooth way as subjects enter into and leave the kernel, as illustrated in supplementary Figure S1. A better approach is to use a window that gradually down weights the influence of subjects in the estimate as subjects move away from the prediction point at *q*_*i*_. A smoother, improved reference class forecasting method uses the Epanechnikov kernel, again defined on the risk quantiles of radius of width *γ* around the subject *i*, with the weight given as:

3 $$ {}w_{k} \,=\, \left\{\begin{array}{ll} \frac{3}{4} \left(1 \,-\, (\frac{|i-k|}{\lfloor{\gamma N}\rfloor})^{2}\right), & \text{for}\quad \!\!\!\!\!i-\!\lfloor{\gamma/N}\rfloor \!\leq k \leq i + \lfloor{\gamma/N}\rfloor\\ 0, & \text{otherwise} \end{array}\right.  $$

We use the same convention at the edges of the risk distribution for the subjects *i*<⌊*γ*/*N*⌋ and *i*>*N*−⌊*γ*/*N*⌋. Under an Epanechnikov reweighting scheme, the weights slowly decay as a function of the distance from the *i*th datapoint. Both the boxcar and Epanechnikov kernels, centred around the 25% risk quantile, are illustrated in Fig. 1.

These ‘local’ reference class forecasting methods are symmetrical around the prediction for the subject *i*; i.e. they use an equal number of datapoints each side of *i*. However, they both can be adapted so that the bandwidth varies, exploiting the maximum possible information around the subject *i* and preserving symmetry. For example, at the median risk quantile, a varying bandwidth method would use all the data. We denote these maximal bandwidth local reference classes, and define the size of the window of information around the *i*th subject as min(*i*,*N*−*i*), where the parameter *γ* now specifies the minimal value that this window can take.

### Estimation of a risk-based CATE using reference class forecasting with exponential tilting

Local reweighting schemes provide a principled approach for determining an ITE for a given subject in the trial up to a certain accuracy, with a certain bias-variance trade-off (see the next section). A different goal is to estimate population ATEs but in populations with a different risk distribution to that of the original trial. Often external populations for which the intervention is intended may differ to those of the trial due to issues such as non-representative inclusion criteria, selection bias or geographical clustering. We denote the estimation of the expected effect under a different population as a conditional average treatment effect (CATE). One interesting, and identifiable, external population can be made by tilting the original sample set through reweighting the contribution from each subject. Exponential tilting of the population weights is one example. Under this scheme we can consider estimating the ATE in an external tilted population that contains more higher risk subjects (or more lower risk ones) as compared with the original trial. In this scheme the *i*th subject with baseline covariates *x* is attributed a weight proportional to *e*^*λQ*(*x*)^ in the estimate of the external ATE, where the free parameter *λ* determines the overall effective sample of the scheme and how far ‘tilted’ the weights are to the highest risk subjects (*λ*>0) or the lowest risk subjects (*λ*<0). The choice of *λ*=0 recovers the original ATE. The ratio of the relative weight *w*_1_ (the lowest risk subject) to the relative weight *w*_*N*_ (the highest risk subject) is thus *e*^−*λ*^. This is akin to estimating the ATE in a population whose participants are recruited with probability *e*^*λQ*(*x*)^ relative to the original trial population.

A CATE could be directly targeted at a population of interest, for example, the set of all screened but excluded subjects (e.g. exclusion due to co-morbidities). One could directly construct the set of weights that target this external population, as the internal validity of the RCT may not apply to the excluded population. We can target the external population by selecting a set of weights such that the weighted distribution of risk in trial participants approximates as best possible the distribution of risk in the external population.

### Effective sample size and bias-variance trade-off

Reference class estimators using reweighting schemes—whether they are global or local—provide unbiased estimators of the targeted treatment effect in the ‘local’ or ‘tilted’ population, but have increased variance with respect to that of the ATE estimated from the original RCT. This is a consequence of the reduced effective sample size within the reference class. The effective sample size can be thought of as the number of subjects (each given weight 1) required to obtain the same accuracy of estimation as in the reweighted population. As a function of the weights *w*_*i*_, the effective sample size is given by $(\sum _{i=1}^{N} w_{i})^{2} / \sum _{i=1}^{N} w_{i}^{2}$. The effective sample size is equal to *N* when all weights *w*_*i*_ are equal to 1 and is strictly less than *N* otherwise. The effective sample size is directly related to the power to detect HTEs using a reweighted reference class. The more distinct the class, the lower the effective sample size, and thus the lower the power to reject the null hypothesis for any given HTE size.

For local reference classes, the effective sample size decreases with decreasing bandwidth of the kernel. This relates to a bias-variance trade-off in estimating the ITE at a reference quantile. The more localised the kernel, the lower the bias to estimate the target ITE, but the greater the standard error of the estimate, which is a function of the square root of the effective sample size. For instance, a kernel that only includes *x*_*i*_ at a reference point has zero bias for the unique ITE but infinite variance of the estimate, as only one outcome is observed.

### Properties of reweighting schemes under no heterogeneity

It is interesting to note that, under an assumption of ‘no treatment effect heterogeneity’, any weighted average of the outcomes is an unbiased estimator of the ATE, albeit with increased variance. If we consider the event ‘no HTE’ as a null hypothesis, then, under this null, the reweighted reference class ITEs will be distributed around the ATE with a variance determined by the effective sample size. This provides for a formal testing framework able to reject this null hypothesis at a certain level of significance, *α*, should there be an HTE under an alternative hypothesis.

## Case study: the HTE of parenteral artesunate for the treatment of severe falciparum malaria

Severe falciparum malaria is a medical emergency characterised by potentially lethal vital organ dysfunction. Mortality is high even in the presence of effective treatment and is strongly dependent on the number and the severity of complications at presentation. Severe malaria represents a spectrum of illness where risk of death is highly predictable from baseline hospital admission covariates. The best prognostic covariates are the presence of coma, the concentrations of base deficit and blood urea nitrogen and also the total parasite biomass [15]. The operational definition of severe malaria as given by the World Health Organisation (WHO) [16] provides cutoffs that allow for efficient triage of patients at the highest risk of death, and standardisation of clinical studies of novel interventions in this clinically important subgroup. However, due to the multifactorial nature of the illness, estimating HTE within this subgroup is well suited for a multivariable risk modelling approach.

A key recent advance in the last decade in the treatment of severe malaria has been the introduction of parenteral artesunate. This has been shown to reduce mortality by up to 30% compared to parenteral quinine [17, 18]. In this section we illustrate our approach to reference class forecasting using the single largest study ever conducted in severe malaria, which compared artesunate to quinine in African children (AQUAMAT) [18]. The results of this trial led to parenteral artesunate becoming the WHO recommended treatment worldwide for severe falciparum malaria. In endemic countries where there is currently no evidence of widespread clinically significant artemisinin drug resistance (i.e. everywhere except Southeast Asia [19]), there is no a priori reason to believe that there are any severe malaria patients who do not benefit from artesunate over quinine. However, characterising heterogeneity in the treatment effect of artesunate is important for understanding the unique pharmacodynamics underlying its superiority. This is especially important in light of emerging resistance to the artemisinin derivatives in Southeast Asia [20]. Characterising the relationship between baseline risk and treatment effect also provides a rational approach for defining study inclusion criteria in order to maximise power at a given sample size.

The following illustrates our suggested approach to assessing HTEs from RCT data and showing how this heterogeneity should be graphically visualised with the help of a multivariable risk model. We first constructed a multivariable risk model of death from severe malaria using data from more than 4000 patients from multiple randomised and observational studies (mostly Asian adults) [17, 21–27]. For all patients in this training dataset, risk of in-hospital mortality was then estimated using a mixed-effects logistic regression model, with the presence of coma (yes/no) and the base deficit concentration (mEq/L) as fixed effects. Study code and country of patient recruitment were added as random effect terms. This model was then used to predict the baseline probability of death in all patients (*n*=5483) recruited in the AQUAMAT study. The assigned risk of outcome was highly predictive of death in the AQUAMAT study (see supplementary Figure S2).

**Fig. 2:**
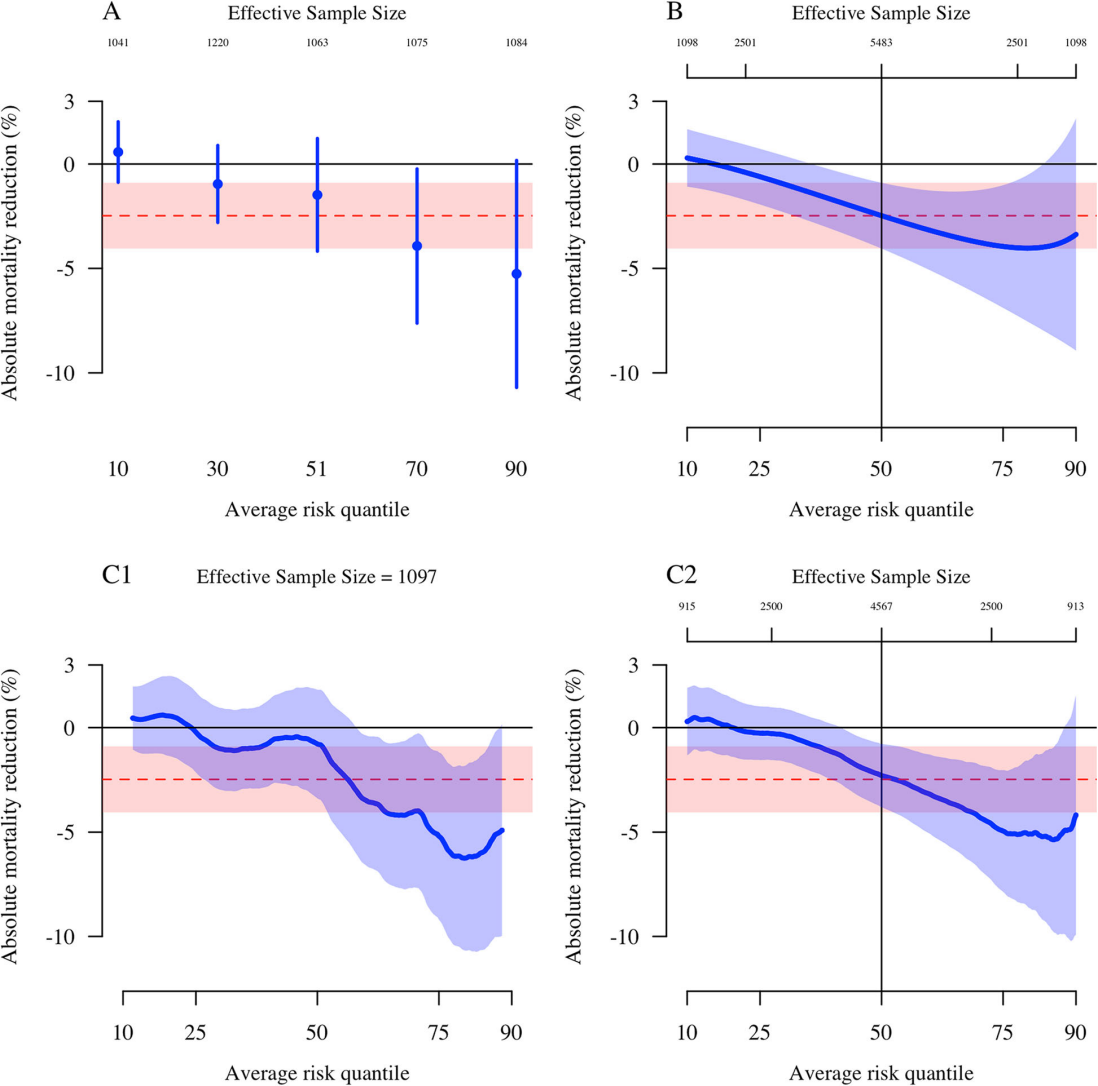
A graphical comparison of four approaches to reference class forecasting of ITEs (*thick blue lines* with the pointwise 95% confidence intervals [CIs] shown as *shaded blue areas*) for patients enrolled in the AQUAMAT study [18]. In each panel the ATE (95% CI) from the original trial (*n*=5483) is shown by the *dashed red line (red shaded area)*. The *left column* shows fixed bandwidth predictors (fixed effective sample sizes approximately equal to one fifth of the original sample size), and the *right column* shows varying bandwidth predictors (varying effective sample sizes). **a** Risk-based quintile partitioning. This does not interpolate between average risks in each subgroup. There is some minor variation in effective sample size due to ties in the multivariable risk scores. **b** Exponential tilting with free parameter *λ* as a global reweighting scheme with varying effective sample size (top *x*-axis). This is centred around the overall treatment effect corresponding to the value *λ*=0. **c1** Epanechnikov kernel with fixed bandwidth chosen for an effective sample size of *n*=1097 (20% of the original sample size). **c2** Epanechnikov kernel with maximal bandwidth reference class. Note that the 50% risk quantile has an effective sample size reduction of 17% with respect to the original trial sample size due to the decay in weights. This contrasts with panel **b**, where the ITE prediction at the 50% empirical risk quantile equals that of the ATE

The original publication of the AQUAMAT study results reported no significant effect from a Mantel-Haenszel analysis of the predefined subgroups [18]. This is unsurprising, as it is well accepted that assessing HTE using single patient covariates lacks power and is prone to false positive results [3, 5]. Using reference class schemes, however, Fig. 2 shows that it is visually apparent that treatment effect is strongly dependent on the baseline risk of death. Panel a in Fig. 2 shows a standard quintile subgroup plot such as that recommended in [4]. Each subgroup has an effective sample size equal to approximately only one fifth of the original sample size, but the two highest quintiles of risk both approximately reach significance at the 5% level in terms of absolute mortality reduction. When using reference class forecasting that interpolates between all risk quantiles, the trend between baseline risk and treatment effect becomes clearer. For example, panel b shows the variation in CATE when varying the distribution of baseline risk using exponential tilting. The estimation of ITEs using an Epanechnikov kernel with varying bandwidth gives very similar results (panel c2).

## Discussion

Randomised trials are designed and powered to estimate ATEs. However, the distribution of baseline risk is often highly skewed. In this case, the ATE will both overestimate and underestimate the benefit of treatment for subjects who have lower or higher than average risk. A consequence of this variation in baseline risk and its influence on the reported treatment effect is that ATEs can be misleading when used to guide treatment recommendations at the individual level. It has been recommended that all trials report how treatment effect varies as a function of baseline risk [4], and the methods described here provide a principled framework for the graphical visualisation of HTEs and the estimation of ITEs and CATEs. Visualisation of HTEs using baseline risk is an important example of the broader concept of constructing an a priori reference class on which to explore heterogeneity.

The classical approach to the estimation of ITEs is to construct a parametric model of the outcome conditional on each possible treatment assignment and observed key subject covariates. Typically this necessitates data transformations of the outcome measurement such as the log-odds scale for logistic regression. Instead, we advocate a two-stage approach, first fitting a parametric model between the outcome and the key subject covariates, preferably from an external data source: this is a multivariable risk model which does not involve counterfactuals. This risk model can then be used to construct causally valid weighted treatment effects which can be directly interpreted in terms of risk-dependent ITEs and CATEs. In brief, instead of modelling the treatment effect directly, we recommend to model the risk and then use classical tests on randomised data to estimate ITEs. A major advantage of this approach is that it provides a formal approach to the use of prior observational data when evaluating the results of a randomised trial.

Graphical visualisation of RCT data using a well-defined risk-based reference class forecasting method such as exponential tilting of subjects’ ‘information weight’ allows for a clear presentation of heterogeneity in treatment effect as a function of baseline risk. These reference class forecasting plots are defined on the scale of the original estimand and are explicitly centred around the ATE targeted by the original RCT, clearly showing the loss in effective sample size as one attempts to predict in the tails of the risk distribution. Alternative methods, such as smoothing splines to estimate the ITEs, add extra complication to the analysis, for example, requiring a transformation of the outcome measure to ensure that the spline estimates remained in the appropriate range (for example, non-negative risk). In addition, they would not be centred around the ATE without considerable modification.

## Supplementary information

**Additional file 1** Supplementary figures.

## Data Availability

The data that support the findings of this study are available from the Mahidol Oxford Tropical Medicine Research Unit, but restrictions apply to the availability of these data, which were used under licence for the current study, and so are not publicly available. Data are, however, available from the authors upon reasonable request and with permission of the Mahidol Oxford Tropical Medicine Research Unit data-sharing committee.
