# Correction to: Graphing and reporting heterogeneous treatment effects through reference classes

**DOI:** 10.1186/s13063-020-04782-5

**Published:** 2020-10-20

**Authors:** James A. Watson, Chris C. Holmes

**Affiliations:** 1 Mahidol-Oxford Tropical Medicine Research Unit, Faculty of Tropical Medicine, Mahidol University, Bangkok, 10400 Thailand; 2 Centre for Tropical Medicine and Global Health, Nuffield Department of Medicine, University of Oxford, Oxford, UK; 3 Department of Statistics, University of Oxford, Oxford, UK

**Correction to: Trials 21, 386 (2020)**

**http://orcid.org/10.1186/s13063-020-04306-1**

Following publication of the original article [1], we were notified that Eq. (1) is wrong and needs replacing. Eq. 1 should have been written:

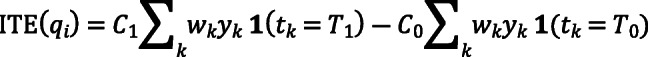



This has divided the sum into its two component parts and replaced the symbol C in the previous version with C_1_ and C_0_.

Instead of: , the definition of C_1_ and C_0_ is given by:  for j=0,1.

In addition, in Eq. (2) and at multiple instances after, γ/N needs to be replaced by γN (there should not be a division symbol).

## References

[1] Watson (2020). Graphing and reporting heterogeneous treatment effects through reference classes. Trials.

