# Supplementary figures and images for: Graphing and reporting heterogeneous treatment effects through reference classes
Source: Trials. 2020 May 7;21:386. doi: 10.1186/s13063-020-04306-1 (PMC7204233; doi:10.1186/s13063-020-04306-1)

## Supplementary figures

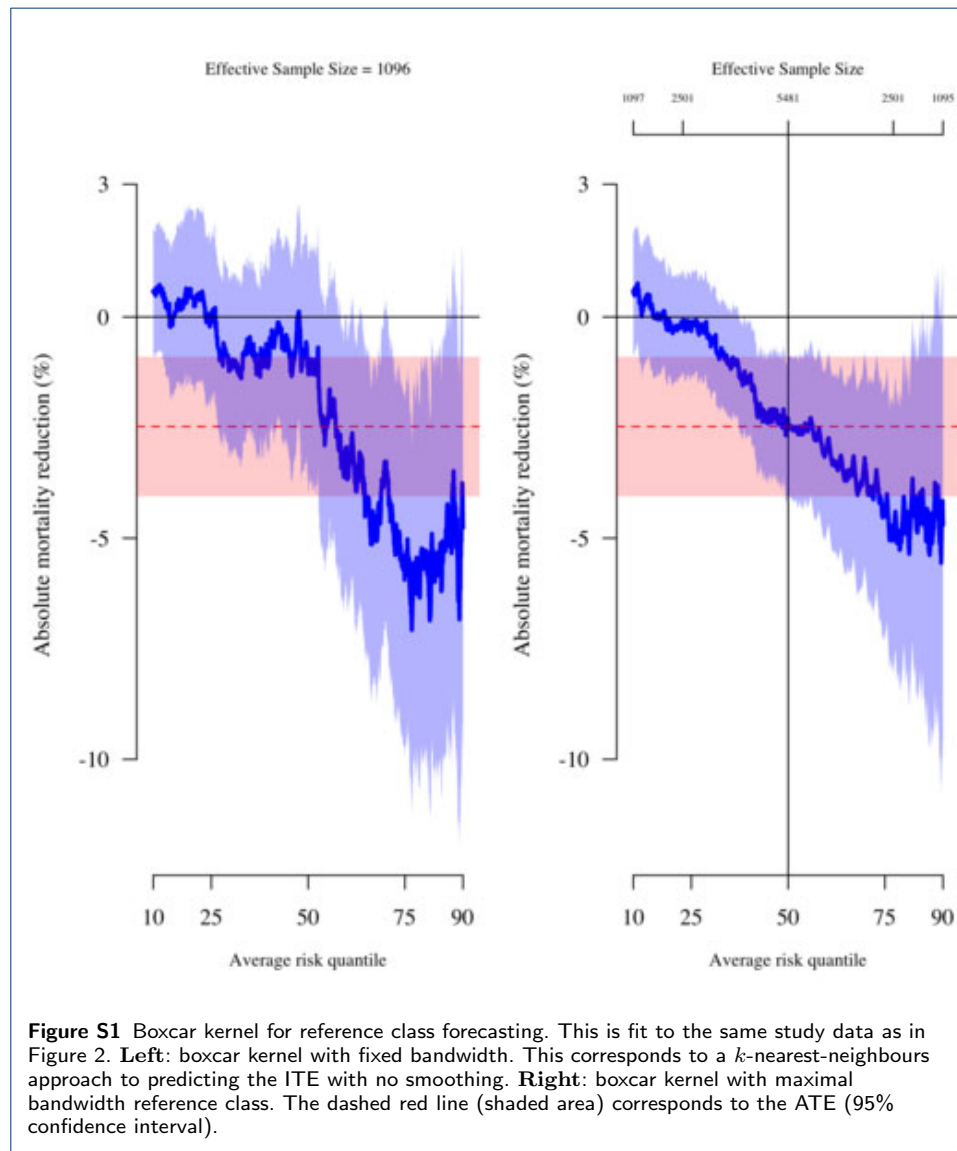

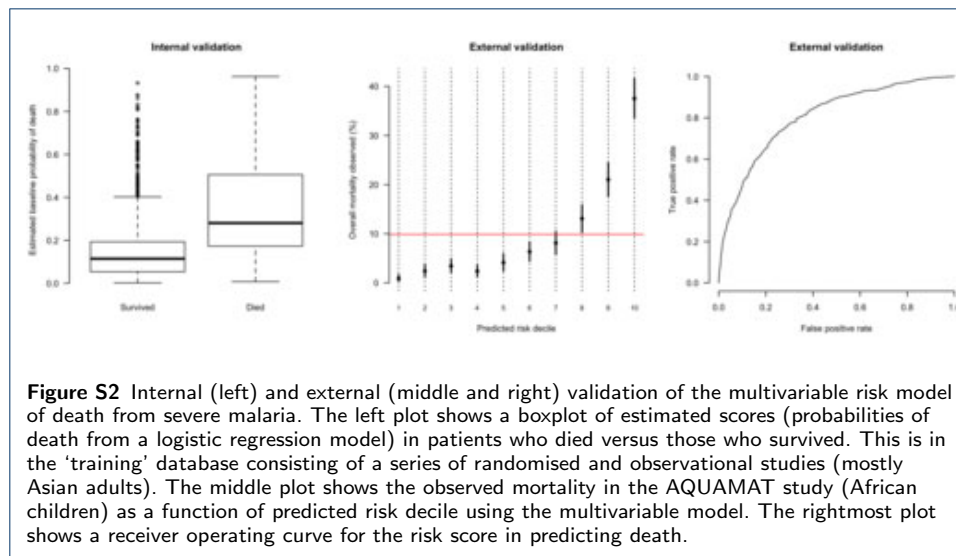

Supplement: Supplementary file 1 — Additional file 1 Supplementary figures. [file 13063_2020_4306_MOESM1_ESM.pdf]
